# Reversible Autoimmune Cardiomyopathy Secondary to a Vaccine-Induced Multisystem Inflammatory Syndrome

**DOI:** 10.7759/cureus.25170

**Published:** 2022-05-20

**Authors:** Ana P Urena Neme, Elmer R De Camps Martinez, Constangela Matos Noboa, Miguel A Rodriguez Guerra, Pedro Ureña

**Affiliations:** 1 Cardiology, Medicina Cardiovascular Asociada, Distrito Nacional, DOM; 2 Medicine, Montefiore Medical Center Albert Einstein College of Medicine, Bronx, USA; 3 Interventional Cardiology, Medicina Cardiovascular Asociada, Distrito Nacional, DOM

**Keywords:** reversible autoimmune cardiomyopathy, covid vaccination booster, multisystem inflammatory syndrome by vaccination (mis-v), multisystem inflammatory syndrome (mis), sars-cov-2

## Abstract

The Dominican government started an early booster protocol, including a heterogeneous vaccination sequence needed based on availability. We report a case of a 25-year-old male who presented with jaundice, and vomiting for 6 days, associated with maculopapular rash (Mucocutaneous features), elevated pro-B-type natriuretic peptide (pro-BNP), erythrocyte sedimentation rate (ESR), transaminitis (> 1000 U/L), thrombocytopenia, echocardiogram evidenced stigmata of heart failure after his third dose of the severe acute respiratory syndrome coronavirus 2 (SARS-CoV-2) vaccination. He was started on steroids and immunoglobulin therapy for multisystemic organ failure syndrome. A significant improvement was noticed, then was discharge; in the post-discharge clinic, he was asymptomatic, inflammatory markers improved, and the echocardiogram showed a recovered ejection fraction. An accurate anamnesis, including a proper chronologic gathering of the events, is essential to recognize a vaccine-multisystem inflammatory syndrome; its prompt assessment and therapy would directly improve the outcome.

## Introduction

The Dominican Government announced the application of the third dose of the severe acute respiratory syndrome coronavirus 2 (SARS-CoV-2) vaccine in June 2021 empirically without a studied protocol because of the peak of the disease despite previous vaccinations.The plan was to use different pharmaceutical brands for every citizen, if needed, after one month of their last dose [1,2]. Several underdeveloped countries have also adopted that preventive strategy empirically. This is a case of a patient with reversible autoimmune cardiomyopathy secondary to a vaccine-induced multisystem inflammatory syndrome after a heterologous SARS-CoV-2 messenger RNA (mRNA) vaccine after two doses of Sinovac-CoronaVac SARS-CoV-2 vaccine (Sinovac Biotech, Beijing, China).

## Case presentation

A previously healthy 25-year-old Hispanic male presented to the emergency department (ED) with a chief complaint of vomiting and watery diarrhea for six days after receiving the third dose of SARS-CoV-2 vaccine with the Pfizer-BioNTech mRNA SARS-CoV-2 vaccine. He received two doses of Sinovac-CoronaVac SARS-CoV-2 vaccine three months prior. Later, the patient added a history of myalgias, muscle cramps, and a fever of 38.5°C (101.3°F) during the first 24 hours period after his vaccination. He had an asymptomatic SARS-CoV-2 infection six months ago and denied family history or symptoms prior to his third vaccination. On the physical examination, he appeared acutely ill, with slight conjunctival jaundice and new-onset maculopapular rash on both cheeks; a blood pressure of 120/70 mmHg, a heart rate of 145 bpm, and a temperature of 38°C (100.4°F). Non-tender cervical adenopathies, a lower basal tactile fremitus, and a distended abdomen with tenderness to the deep palpation of the right hypochondrium were found. 

Laboratory evaluation was notable for thrombocytopenia, transaminitis, elevated anti-SARS-CoV-2 immunoglobulin (IgG), hyperbilirubinemia, elevated B‐type natriuretic peptide (BNP), and D dimer (Table 1). 

**Table 1:** Laboratory results of the patient during admission AST:  aspartate transaminase, ALT: alanine transaminase,  SARS-CoV-2: severe acute respiratory syndrome coronavirus 2; IgG: immunoglobulin;

Tests	Results	Reference range
Platelets	123,000	150,000-450,000/ μL
Alkaline phosphatase	92	0-115 U/L
AST	630	0-41 U/L
ALT	5,600	0-40 U/L
Anti-SARS-CoV-2 IgG quantitative	37,000.0	
D-dimer	2,270	0-500 ng/dL
Procalcitonin	0.42	< 0.5 ng/mL
Total bilirubin	4.68	0-1.1 mg/dL
Direct bilirubin	3.46	0.00-0.25 mg/dL
Indirect bilirubin	1.22	0-0.8 mg/dL
Pro-B-type natriuretic peptide	1,055	pg/mL
Urea	10	15-39 mg/dL
C-Reactive Protein	4.69	6.9-12.2 ng/dL
Erythrocyte sedimentation rate	25	Less than 15 mm/h
Creatinine	0.92	0.92 mg/dL
Troponin	<0.10	0-0.3 ng/mL
Creatine kinase-MB	6	6 U/L
Alpha-1 antitrypsin	122.10	90-200
Hepatitis C Antibody	Negative	
Epstein-Barr Virus Antibody	Negative	
Entamoeba histolytica	Negative	
Leptospira Antibody	Negative	
Dengue antibody	Negative	
Rheumatoid factor	Negative	
C3	75.58	higher than 87
C4	13.20	above 19

**Figure 1:**
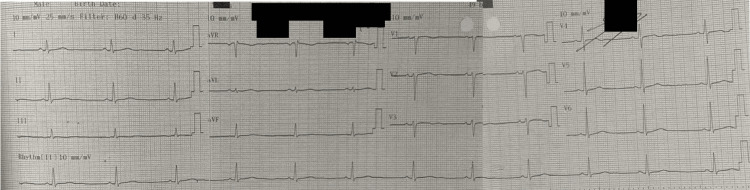
Electrocardiogram (ECG)

On admission, the electrocardiogram (ECG) showed resolution of the tachycardia after the use of steroids and immunoglobulins with a heart rate of 50 bpm, a PR of 160 milliseconds (ms), without ST-T segment alterations, and a QTc of 457 ms (Figure 1).

The abdominal ultrasound reported a bilateral pleural effusion, ascites, acalculous cholecystitis, and a grade 2 hepatic steatosis without signs of portal hypertension. He was admitted to the hospital, the day after his admission, while he was sleeping, the patient developed non-radiated, oppressive midsternal chest pain, rated 6 out of 10 on a pain scale, associated with dyspnea and palpitations.

Cardiology was consulted due to acute chest pain, dyspnea, and D-dimer elevation; angiotomography for pulmonary emboli (PE) was negative. A transthoracic echocardiogram showed generalized hypokinesia of the left ventricle with an ejection fraction of 41% (Table 2). The patient had a negative infectious workup, and no alternative etiology of presumptive myocarditis was identified. A cardiac magnetic resonance imaging showed normal biventricular volumes, morphology, and systolic function, with no signs of myocardial fibrosis.

**Table 2:** Echocardiographic measurements TAPSE: Tricuspid annular plane systolic excursion

	Value	Unit
Aortic Root	25	mm
Left Ventricle	48-37	mm
Ejection fraction	41	%
IV Septum	7	mm
Posterior wall	7	mm
Left Atrium volume	17	Mml/m2
Left Atrium	37	mm
Right Atrium	33	mm
Right Ventricle	40	mm
TAPSE	25	mm
Pulmonary Artery	25	mm
Inferior Vena Cava	1.9	gr/m2

After ruling sepsis out, the Brighton Collaboration network criteria to identify "Multisystem Inflammatory Syndrome in Children and Adults (MIS-C/A)" in the evaluation of adverse events following immunization were used. The patient fulfilled the following criteria: presence of fever for more than three consecutive days, musculocutaneous and gastrointestinal manifestations, elevated erythrocyte sedimentation rate (ESR), and pro-B-type natriuretic peptide (pro-BNP), thrombocytopenia, physical stigmata of heart failure, and echocardiographic findings after vaccination against SARS-CoV-2. Intravenous immunoglobulins infusion and methylprednisolone were started for this diagnosis.

On the third day of admission, the patient presented a blood pressure of 150/90 mmHg; amlodipine 5 mg was started for glucocorticoid-induced hypertension. The patient responded well to the therapy, and his transaminases started to downtrend (Table 3).

**Table 3:** Transaminases trending during hospitalization AST: aspartate transaminase, ALT: alanine transaminase

	Daily trending of transaminases						Reference Range
ALT	5600	2400	2135	1940	1940	1460	0-40 U/L
AST	630	162	160	64	64	51	0-41 U/L

The patient was discharged on carvedilol 6.25 mg, lisinopril 5 mg, dapagliflozin 10 mg, and prednisone 20 mg daily for 14 weeks with a tapering protocol. At the discharge clinic, continuous follow-up was given, three months after his discharge liver function tests trended down to within normal limits and symptomatic improvement was evident. An echocardiogram reported a recovered ejection fraction of 54% (Table 4). One month after the heart function improvement, guideline-directed medical therapy for heart failure was de-escalated. 

**Table 4:** Echocardiographic measurements on follow-up TAPSE: Tricuspid annular plane systolic excursion

	Value	Unit
Aortic Root	26	mm
Left Ventricle	49-32	mm
Ejection fraction	54	%
IV Septum	8	mm
Posterior wall	7	mm
Left Atrium volume	15	Mml/m2
Left Atrium	32	mm
Right Atrium	31	mm
Right Ventricle	29	mm
TAPSE	20	mm
Pulmonary Artery	22	mm
Inferior Vena Cava	12	gr/m2

## Discussion

Multisystem inflammatory syndrome (MIS) is an uncommon condition that could worsen the outcome in COVID-19 patients. However, there is a post-COVID-19-MIS established in the literature; it usually manifests four to six weeks after the acute phase of the infection, but there is literature exposing the risk period up to sixteen weeks [3,4]. In April 2020, this syndrome was recognized in the United Kingdom (UK) and named Pediatric Inflammatory Multisystem Syndrome ( i.e., Multisystem Inflammatory Syndrome in Children - MIS-C) [5,6]. Subsequently, there were reports of a similar syndrome in adults (Multisystem Inflammatory Syndrome in Adults - MIS-A) [7,8]. In both cases, multiple organ systems, including the heart, get affected as other significant complications; the cytokine storm triggers this inflammatory syndrome in this viral infection [9].

A new multisystem inflammatory syndrome has been described. It corresponds to SARS-CoV-2 vaccination (MIS-V) [10]; it is an uncommon entity described in the literature, less common than the MIS, MIS-C, or MIS-A [11]. The exact epidemiology of the MIS-V is still unknown. A possible hypothesis based on the cytokine storm and the immune hyper-reactivity is suggested, although an asymptomatic covid infection close to the date of the vaccination could lead to an MIS representing a possible bias on the diagnosis. This entity is a ruled-out diagnosis with elevated coagulopathy and inflammatory markers. The heart's affection could also be present as an autoimmune cardiac reaction leading to a depression of its function and fatal arrhythmias [12].

The autoimmune myocarditis (AIM) mechanism is not entirely understood, but the flare of autoimmune disorders classically triggers it [13]. As an autoimmune reaction, the MIS-V could lead to AIM, most probably due to the cytokine storm causing an inflammatory reaction by the hyper-reactivity of the immune system to the healthy cardiac tissue. The therapy for AIM is based on the patient's symptoms, but in severe cases, immunosuppression is required to improve cardiac function and avoid a potential risk of death [14].

The management of MIS-V is based on the guides for the treatment of MIS-C and MIS-A; supportive measures and immunomodulatory treatment are crucial to the patient's outcome. Immunosuppressive therapy used is steroids and immunoglobulins [15]. The steroid is the first-line therapy to suppress the immune and inflammatory process; its mechanism is based on the affinity to a specific receptor on the membranes to modify transcription, affecting protein synthesis; this therapy also inhibits the phospholipase A2 [16,17]. The immunoglobulin (IVIG) is usually used when the patient does not improve with steroids or when the patient is severe or critically ill; it reduces the inflammatory activity, decreasing the stress in the coronaries in a patient with vasculitis, but in patients with MIS-C, it has shown a positive response [18]. 

In the literature, we found only three cases illustrating the MIS-V post-SARS-CoV-2 vaccine, typically after mRNA SARS-CoV-2 vaccine such as the Pfizer-BioNTech one and after the Oxford/AstraZeneca SARS-CoV-2 vaccine [19,20]. 

We could not find any data about MIS-V after the third dose in patients immunized with the Sinovac-CoronaVac SARS-CoV-2 vaccine, nor the evidence of safety or efficacy of this heterologous SARS-CoV-2 vaccine combination. An accurate anamnesis, including a proper chronologic gathering of the events, is essential in medicine to establish the best approach for our patients. Further investigations are needed to safely establish transversal data and a universal protocol to vaccinate our patients against COVID-19, avoiding potential complications and clarifying the difference between MIS and MIS-V which in some cases could be a matter of discussion. Our report is an exceptional case of an MIS-V induced by the third dose of SARS-CoV-2 vaccine in a post-COVID-19 patient who coursed with thrombocytopenia, elevated ESR, transaminitis, and new heart failure onset less than 4 weeks of his third booster shot, and was successfully treated with immunosuppressive therapy.

## Conclusions

Reversible autoimmune cardiomyopathy induced by vaccine-induced multisystem inflammatory syndrome could be caused by the third dose of a heterologous SARS-CoV-2 vaccine. Our patient is an interesting and unique case of reversible cardiomyopathy due to MIS-V. Further investigations are needed to safely establish transversal data and a universal protocol to vaccinate our patients against COVID-19, avoiding potential complications.
